# 'Operating in the dark’: perspectives of health service users and healthcare workers on quality of diabetes care at a public referral hospital in Blantyre, Malawi - a qualitative study

**DOI:** 10.12688/wellcomeopenres.21374.1

**Published:** 2025-05-16

**Authors:** Chimwemwe Kwanjo Banda, Belinda Thandizo Gombachika, Moffat Nyirenda, Adamson Muula

**Affiliations:** 1 School of Global and Public Health, Kamuzu University of Health Sciences, Blantyre, Malawi; 2 Public Health group, Malawi Liverpool Wellcome programme, Blantyre, Malawi; 3 School of Nursing, Kamuzu University of Health Sciences, Blantyre, Malawi; 4 Uganda MRC/UVRI Research Unit, Entebbe, Uganda; 5 NCD-BRITE Consortium, Kamuzu University of Health Sciences, Blantyre, Malawi; 6 Africa Center of Excellence in Public Health and Herbal Medicine, Kamuzu University of Health Sciences, Blantyre, Malawi

**Keywords:** Healthcare system, primary health care, non-communicable diseases, service accessibility, self-management support

## Abstract

**Background:**

Poor glycemic control and medical complications are common among people living with diabetes in Malawi. The healthcare system is part of an environment in which people with diabetes manage themselves and can act as facilitators or barriers to diabetes self-management. This study aimed to explore the effects of diabetes health service users and healthcare workers (HCW) on the quality of diabetes care at the Queen Elizabeth Central Hospital (QECH) diabetes clinic.

**Methods:**

This qualitative study recruited service users (people with diabetes and family members) and HCW (medical doctors, clinical officers, and nurses) at the QECH diabetes clinic, a public referral hospital in Blantyre. Data saturation was achieved after 20 individual in-depth interviews (IDI) and four focus group discussions with people with diabetes, 20 IDIs with family members of people with diabetes, and eight key informant interviews with HCW. A framework analysis approach was used to guide data analysis.

**Results:**

Participants shared positive and negative perspectives regarding the quality of care. Five major themes emerged: healthcare system organization, drug and treatment equipment supply, pathways to diagnosis, diabetes health education and awareness, and ongoing monitoring, prevention, and management of complications. The participants appreciated free services at the clinic, including glucose checks, health education, and medications. However, many were concerned with understaffing, which led to long clinic waiting times, inadequate patient assessments, and failure to diagnose diabetes complications.

**Conclusion:**

The quality of care in diabetes clinics is perceived to be mixed. Although service users appreciated the available services, they noted other deficits at the clinic, which made it difficult for them to receive necessary care. Good quality diabetes care services are essential for improving the well-being of people with diabetes.

## Plain Language Summary

Approximately 468,000 people live with diabetes (sugar disease) in Malawi. Diabetes mellitus is a condition in which an individual has an abnormally high blood sugar level. People with sugar diseases can control their blood sugar levels to near normal levels by eating recommended healthy foods, exercising regularly, and taking medications appropriately if applicable. When people with diabetes fail to achieve adequate control of their sugar levels, they experience damage to important organs such as the brain, kidney, heart, eyes, and nerves. Good quality care for people with diabetes at health facilities also helps keep the disease under control.

This study was conducted at the Queen Elizabeth Central Hospital (QECH) diabetes clinic, a non-paying referral hospital in Blantyre. Nearly seven out of every ten people attending the QECH diabetes clinic had uncontrolled sugar levels, and five out of every ten had one or more organs damaged due to the high sugar content. The aim of our study was to determine the perceptions of the quality of diabetes care at QECH among people with diabetes, their family members, and healthcare workers.

We interviewed 20 people with diabetes, 20 family members; two medical doctors, two clinical officers and four nurses at the facility. We also conducted four group interviews with people with diabetes (each group having 6-12 people). Participants perceived that care services at the diabetes clinic were good in some areas and not good in others. Many participants were concerned with understanding at the clinic, which led to long clinic waiting times, incomplete patient assessments, and failure to identify diabetes complications. These findings provide an overview of the situation at the QECH diabetes clinic and how it affects care. There is a need to address the identified problems to improve care at facilities to help promote the well-being of people with diabetes.

## Introduction

The number of people living with diabetes mellitus (DM) is projected to increase by almost 20% from 537 million in 2021 to 643 million by 2030 ^1^. As the number of people living with DM increases, demand for quality health services also increases. An estimated 468,000 people live with DM in Malawi ^1^, with a prevalence of 2.2% among those aged 25–64 years ^2^. The Queen Elizabeth Central Hospital (QECH) in Blantyre, a referral and academic hospital in southern Malawi, operates one of the biggest diabetes clinics in the country, with over 4,400 registered, and about 150 people seen per clinic day that runs once a week. Of concern is that many of these patients have poorly controlled sugar levels and suffer from one or more DM complications. A previous study of 620 participants at the QECH diabetes clinic reported that 75% of the clinic attendees had an HbA1c of more than 7.5%, 46.4% had neuropathy, 34.7% had nephropathy, 34.7% had retinopathy, and 7% had a history of stroke as a complication of DM and/or hypertension ^3^. These poor outcomes reflect self-management practices and the quality of the healthcare they receive.

Self-management includes several day-to-day activities related to diet, physical exercise, medication, blood glucose monitoring and foot care ^4– 6^. Due to the chronic nature of the disease, self-management is essential for people living with DM to keep the disease under control and prevent its complications. The lead researcher (CKB) developed an interest in diabetes care from her clinical experience as a nurse educator, where she observed that at any point in time, there was a patient with severe complications of diabetes in medical and surgical wards at the referral hospitals. This led to an assessment of self-management among people with diabetes at the QECH diabetes clinic, which found that only 33% (n = 510) reported following recommended practices on diet, exercise, foot care, and medication ^7^.

In addition to self-management, the quality of care provided to individuals with DM and their families is complementary ^8, 9^. The World Health Organization (WHO) defines quality of care as “the degree to which health services for individuals and populations increase the likelihood of desired health outcomes” ^10^. According to Avedis Donabedian, healthcare quality has three dimensions: structure, process, and outcome ^11^. In the quality of the healthcare model, structure refers to the available physical resources at a facility including infrastructure, equipment, and personnel; process refers to the interaction between clients and healthcare workers (HCW), plus the actual care given; and outcome refers to the results of the care ^11^. Good quality care for people with DM emphasizes self-management support. Self-management support includes a varied range of activities such as intellectual support (patient education and information giving), behavioral support (helping clients set goals for self-management and lifestyle modification), emotional support (counselling), and tangible resources (medication and equipment) ^9, 12, 13^. Previous studies conducted at other public health facilities in Malawi reported suboptimal quality of care for people with DM ^14– 17^. Applying the Donabedian Model, this study aimed to assess the quality of care at the QECH diabetes clinic, as perceived by healthcare workers (HCW) and service users (people with DM and their family members).

## Methods

A cross-sectional qualitative approach was used to collect opinions, beliefs, perceptions, and motivations of participants (people living with diabetes, their family members, and HCW) regarding the quality of care at the QECH diabetes clinic ^18, 19^. The qualitative method was preferred to help understand the perspectives and experiences of the participants regarding the quality of care and how it influences self-management in people with diabetes. We adopted an interpretivism paradigm because we considered knowledge to be subjective and different individuals experienced the same phenomenon differently ^20^. The study conformed to the Consolidated Criteria for Reporting Qualitative (COREQ) study guidelines ^21, 22^.

### Study setting

The study was conducted at QECH, a referral and teaching hospital in Blantyre, southern Malawi. Permission to conduct the study at QECH was sought from hospital management through the head of the medical department, which oversaw the diabetes clinic at the time the data were collected. At the time of data collection, the clinic was operating once a week on Tuesdays, and seeing an average of 150 clients on each clinic day. The staff at the clinic included medical doctors, clinical officers, and nurses. Diabetes Association of Malawi (DAM) members were also present on each clinic day and assisted with weighing clients, checking their Blood Pressure (BP), and ensuring order in the waiting area.

On each clinic day, clients first went to the hospital laboratory for a fasting blood glucose (FBG) test between 5am and 8am. From 8am to 11am, clients assembled at the diabetes clinic waiting area for body weight and BP checking; at the same time, health education was provided by one of the nurses or DAM peer educators. Afterwards, those with diabetic ulcers were dressed by nurses trained in diabetes wound care. From 1pm, FBG results were distributed by clinic clerks and clients triaged according to their blood glucose levels. Those with glucose levels < 70 mg/dL or ≥ 200 mg/dL were prioritized to be seen first by medical doctors or clinical officers in private consultation rooms. Those with glucose levels between 70 mg/dL and 200 mg/dL, were reviewed by nurses in an open space in the clinic’s waiting area. Consultations were started at 1:30 until the last client was seen. After the consultations, the clients went to the clinic clerks for registration and had their prescription stamped as a requirement to obtain medication at the hospital pharmacy. The pharmacy closed at 4 p.m.; therefore, all clients seen beyond that time were required to return the next day to get their medication. Upon collecting medication from the pharmacy, clients returned to the laboratory for a booking of their next date of review. The next appointment date was between three and five months, depending on space, as they only booked up to 150 people per clinic day.

### Participants

The participants were adults with diabetes (aged 18 years and above), adult family members, and HCW at the QECH Diabetes Clinic. We triangulated sources of qualitative data to obtain multiple perspectives and validate the data ^23^. The sample size for each group of participants was determined by data saturation after no new themes emerged because an appropriate sample size in qualitative research adequately answers the research question ^24^. Individuals with diabetes were included if they were aged 18 years and above, had a diagnosis of diabetes mellitus for over six months and were able to communicate in English or Chichewa (a local language, widely spoken in Malawi) languages. Participants were excluded if they were acutely ill or had cognitive impairments. Purposive sampling was used to select participants with the aim of including both type 1 and type 2 diabetes, an equal number of male and female participants, and a mix of people with varying durations of living with diabetes to ensure maximum variation in viewpoints. Twenty individuals with DM participated in individual in-depth interviews (IDI). Notes were made during the interviews to capture the main points. The duration of the interviews ranged from 30 to 71 min. Another 55 individuals with DM participated in the four focus group discussions (FGDs) ( Table 1). Two FGDs were conducted with male participants, and two FGDs with female participants. Separation by gender was made to allow participants to express themselves freely among fellow participants of the same gender. Each FGD included a minimum of six participants and a maximum of 10. The number of participants selected ensured that each FGD had enough people to generate meaningful discussions and not too many such that others could be left out ^25^. The FGDs also explored participants’ self-management practices and their views on the quality of care at the clinic. The duration of FGDs ranged from 57 to 90 min. It was planned that each participant would receive refreshments (a bottle of water, a cup of tea, and brown bread) for their time. However, the nurses at the clinic recommended that instead, the participants should receive an amount of money equivalent to what would have been used to buy the refreshments to avoid disturbing their eating schedule. Therefore, each participant received a K1000 (equivalent to US$1.30 at that time) to compensate for their time. In addition, all participants received a diabetes information brochure for Malawi, which was produced by the DAM to take at home for reading.

**Table 1.** Characteristics of participating people with diabetes.

Characteristic	IDI * (n = 20)	FGD ** (n = 34)	Total (n = 54)
Sex			
Male	10	15	25
Female	10	19	29
Age (years)			
25 – 34	3	0	3
35 – 44	2	8	10
45 or more	15	26	41
Residence			
Blantyre Urban	13	30	43
Blantyre Rural	5	3	8
Outside Blantyre	2	1	3
Diabetes Type			
Type 1 ^#^	4	3	7
Type 2 ^# #^	16	31	47
Treatment			
Diet only	2	0	2
Insulin therapy (requiring insulin injection)	10	6	16
Oral medication	8	28	36

Key: ^*^Individual in-depth interview; **Focus group discussion: ^#^A type of DM where the pancreas produces little or no insulin; ^#^^#^A type of DM where the body fails to use available insulin.

Twenty family members ( Table 2) participated in the IDIs regardless of whether their relationships were interviewed. Family members were included if they were aged 18 years and above, had escorted a person living with diabetes to the clinic regardless of how long the person had diabetes, and were able to communicate in English or Chichewa languages. Family members were excluded if they were accompanied by a sick patient. Convenience sampling was used to recruit family members because only a few of them visited the clinic. Most of the family members visiting the clinic were female; therefore, we did not aim for an equal representation of males and females. Interviews were conducted at the QECH Diabetes Clinic. A structured interview guide was used to ensure that similar issues were explored by all family members regarding how their relationship managed diabetes and their perception of the quality of care at the clinic. The duration of the interviews ranged from 20 to 63 min.

**Table 2.** Characteristics of Participating Family members.

Characteristic	Frequency (n = 20)
Sex	
Male	4
Female	16
Age (years)	
18 – 24	3
25 – 34	4
35 – 44	7
> 45	6
Residence	
Blantyre Urban	17
Blantyre Rural	2
Outside Blantyre	1
Relationship to client	
Spouse	5
Child	7
Parent	2
Grandchild	2
Domestic worker	3
Niece	1

Eight HCWs ( Table 3) participated in Key Informant Interviews (KII). This group of people was selected because of their knowledge and experience working with people living with diabetes. Healthcare workers were included if they had a work experience at the diabetes clinic and were excluded if they were not available at the clinic for any reason during the data collection period. Purposive sampling was used to ensure that a varying mix of health professionals was included to obtain the maximum variation of viewpoints. Interviews were conducted to obtain views and observations of healthcare workers on

**Table 3.** Characteristics of Participating Healthcare Workers.

Characteristic	Frequency (n=8)
sex	
Male	3
Female	5
Age	
26 – 35	3
36 – 45	2
> 46	3
Profession	
Medical Doctor	2
Clinical Officer	2
Registered nurse midwife	1
Enrolled nurse midwife	3
Post qualification training in diabetes management	
Yes	3
No	5
Work experience at diabetes clinic	
< 1 year	2
1 – 5 years	4
> 5 years	2

Table 1– Table 3 are a summary of the characteristics of the participating people with diabetes, family members, and healthcare workers.

### Data collection instruments

Four separate interview guides were developed by the authors based on the literature on diabetes self-management and quality of care ^26, 27^ and were made available as extended data ^22^. The interview guide for IDIs and FGDs for people with diabetes included questions on perceived barriers and facilitators to diet, exercise, medication, foot care, and blood glucose monitoring; perceptions of the care received at the diabetes clinic; and suggestions for improving the care at the clinic. Family members of patients with DM were asked to explain their relationship (the index patient) to keep diabetes under control. Probing was done to obtain more information on diet, medication adherence, foot care, exercise, environmental barriers and facilitators, and suggestions for improvement of care. Healthcare workers were asked to explain their perception of self-management practices for adults living with DM seen at the QECH diabetes clinic; availability of guidelines, protocols, and policies to guide diabetes care; and their role in caring for people with DM at the clinic. Each of the four interview guides was pretested with the respective target population to assess the clarity of the questions; no modifications were suggested by the participants after the pre-test.

### Data collection

The data were collected between November 2017 and June 2018. All interviews were conducted by the first author (CKB) who had training and experience in qualitative data collection; she was a PhD candidate and had a Master of Science in Nursing. Audio recordings of all interviews were obtained with prior permission from the participants. Individuals with DM and their family members were approached face-to-face for recruitment on diabetes clinic days. On each clinic day, before the health education session started, the person providing the education invited the researcher to come to the front. The researcher then introduced herself to the objectives of the study. As the clinic was going on, the researcher observed the activities and interactions between clinic attendees and each other, as well as with healthcare workers, and wrote some field notes. After the clinic morning routines were completed (usually around 10 am), as the clinic attendees were waiting for laboratory results, the researcher approached individuals to invite them for IDI or FGD. If they were interested, they were taken to a private room within the clinic to receive more information about the study. If they were interested in participating, they signed an informed consent form. The IDIs lasted approximately an hour, while the FGDs lasted approximately an hour and a half. The IDIs and FGDs were conducted in a private room where consent was obtained. A research assistant took short notes on the FGDs. All individuals who approached the IDIs and FGDs agreed to participate. However, one participant withdrew from the FGDs because she wanted to be excused and did other things.

Healthcare workers were identified through the heads of the sections. The Chief Nursing Officer for the Ambulatory Services and the Head of Medicine Department provided lists of names for nurses and medical doctors/clinical officers working in diabetes clinics, respectively. The researcher then contacted the individuals in person or via telephone to introduce the study and book an appointment for the interview. Interviews were conducted on an agreed day and time, convenient for the participant, private, within the hospital. Data collection was stopped when there was data saturation, and no new themes were merged.

### Data analysis

Interview records were transcribed verbatim (and translated to English if the interview was conducted in Chichewa). A framework analysis was used to guide the data analysis process to allow an easy audit trial of how themes were developed ^28, 29^. In an iterative process, the transcripts were repeatedly read for familiarization to gain an overview of recurrent themes from the data, and notes on the themes were written down. A thematic framework was developed based on emergent themes from the data and the Donabedian model of QoC. Initial indexing (or coding) using the descriptive textual system was performed manually by the first author. During this step, the transcripts were read and textual indexing references (codes) were recorded on the margins as comments in Microsoft Word. After indexing, charting was performed, where summaries of data from each transcript were made and fitted into the corresponding box in the thematic framework created in Microsoft Excel to organize and make sense of the extensive data material. The codes were refined inductively by comparing each new piece of data with previously coded data to identify the patterns and differences. Finally, mapping and interpretation of the data were performed based on the agreed themes. The analysis was conducted by CKB, LK (who was a senior lecturer, Community Health Nursing Department, School of Nursing, Kamuzu University of Health Sciences) and DJ (who was an Associate Professor, Mental Health Nursing Department, School of Nursing, Kamuzu University of Health Sciences).

### Maintaining rigour

To ensure rigor of the qualitative process, four criteria for trustworthiness (credibility, dependability, confirmability, and transferability) were used ^30^. Credibility was maximized by conducting all qualitative interviews conducted by the principal investigator to ensure consistency in the interview technique and to allow deep probing. Additionally, member checking (participant validation) was performed, whereby people with diabetes who participated in IDIs and FGDs were reached out through the diabetes clinic and were given copies of the transcripts and identified themes for content validation; all agreed that it represented their perceptions. Individuals with diabetes or their family members were approached in person through the QECH diabetes clinic when they came for review to participate in member checking. Clinical records were used to identify participants who had come on that particular day. For each FGD, two participants were used for member checking. HCW were approached in person or by email to participate in member checks. As data collection progressed, ongoing debriefing sessions were conducted with the physician and nurse in charge of the diabetes clinic, research supervisors (ASM, MJN, BTG), and the executive members of the diabetes association of Malawi to confirm emergent themes.

An audit trial was conducted to ensure the dependability of the research process and the transferability of the data. Records of recruitment, sites for data collection, duration of interviews, and how data codes were developed were kept. Triangulation of data sources (people with diabetes, family members of people with diabetes, and healthcare workers from diabetes clinics) and data collection methods (IDIs and FGDs) was performed to minimize researcher bias and promote confirmability ^31^. The codes, categories, and themes were discussed and agreed upon by CKB, LK, and DJ.

### Findings

The participants had mixed views on the quality of care at the QECH Diabetes Clinic. Five major themes emerged from the data and were categorized according to the three dimensions of the Donabedian model of quality of care ^32^ Table 4).

**Table 4.** Main themes and categories.

Dimension of care	Theme	Categories
**Structure**	Healthcare system organization	• Accessibility of services • Shortage of staff • Long waiting time • Lack of diabetes primary healthcare services • Sustainability of programs
	Drugs and treatment equipment supply	• Medication and insulin syringes stock outs • Medication dosages
**Process**	Pathways to diagnosis	• Delayed diagnosis • Fragmentation of care • Mismanagement of diabetes
	Diabetes health education and awareness	• Importance of health education sessions • Absence of diabetes education protocols • Lack of community awareness initiatives • Peer education
**Outcome**	Ongoing monitoring, prevention and management of complications	• Glucose monitoring • Screening for and management of complications • Psychological support

***Theme 1: Healthcare system organization.*** Accessibility of diabetes services was perceived to be a challenge for all groups of participants due to the centralization of the diabetes clinic at the central hospital at the time the data were collected.

*“although most of the patients come from within Blantyre, there are others who come from outskirts of the district and outside. Some come from Chileka, from Kunthembwe, others from Chiradzulu district and others even from Zomba.” **HCW2, Female, 62*** **years**

The diabetes clinic at QECH was therefore often congested with fewer HCW, leading to delays in service provision. Many participants expressed concern over the time it took to complete all clinical activities.

*“They have to come at 5.00am. Actually, they stop everything at home, even at work it’s hectic and they are only back after 5.00pm and the pharmacy is closed.” **HCW4, Male, 45 years***
*“People leave their homes as early as 4am, maybe 5am. Some came here yesterday evening and have slept here at the hospital waiting for today’s clinic **.” person with diabetes 19, Male, 61 years***
*“The only thing that worries me about this place is that we have to come early morning, and we go back in the evening. This is what I find to be a big problem.” **FM4, female, 34 years***

In particular, people with diabetes and family members expressed frustration with the flow of diabetes clinic services, which took about the whole day for them to access the service. Participants had some suggestions on how services could be restructured to manage overcrowding and minimize delays.

*“No, we shouldn’t be coming again in the afternoon, time is money. Once we are done with blood testing, they should give us our medication prescriptions, we collect our medicine and go. Only those coming for the first time should remain, since they are newly starting the process. We shouldn’t be treated the same way? To say the truth, this hurts.” **P10FGD1, Female, 63 years***
*“I also feel that the time that people with diabetes spend here is very long. Take for example, my wife and I came here around 5 am, do you understand? That means automatically we will also leave this place around 5pm... if only that time was made short. We have many things to do.” **FM20, Male, 48 years***
*“The results should come out quickly. Imagine people left their homes 3 am, and at the moment we are doing nothing waiting for 1 pm, then we will stay up to past five before we leave.” **P1FGD3, Male, 45 years***
*“You know, the number of people with diabetes is continuously increasing. I would have loved if health centers were empowered, so that diabetes clinics are conducted at the health centers. Whether personnel from QECH will go to the health centers or it will be people from the health centers. There are doctors in the health centers. But if they did a clinic even once a month, it would reduce the numbers. Because QECH it is overcrowded. So, if services were extended to health centers, it would help, and if it were extended to district hospitals, it would also help. Because some people are coming from as far as Chikwawa, some from Mulanje… they come from faraway places. Some sleep here so that the next day they can attend the clinic. So, if district hospitals and health centers were strengthened, it would help. The way they have done with management of HIV in smaller health facilities, if they did the same for diabetes. Diabetes and BP because they go together, it would help so much.” **person with diabetes 01, Female, 67 years old.***

Participants stated that efforts were made to introduce diabetes clinics at the primary care level in the Blantyre District Health Office (DHO); however, the clinics have not been sustained.

*“Although arrangements were made through the DHO [District Health Office] to introduce diabetes clinics at health centers and reduce congestion at QECH, it only happened may be for a month. When people went there, they would be told ‘the doctor or nurse for that [diabetes] is not available”. So, people started flocking back here at QECH… if only those clinics were sustained in the health centers **.” person with diabetes 19, Male, 61** **years***

***Theme 2: Drug and treatment equipment supply.*** People with diabetes appreciate the free provision of diabetes medications in public hospitals. However, the availability of medication was not consistent at any public facility, which consequently necessitated out-of-pocket purchase of medication from private pharmacies. This is what one of the healthcare workers had to say.

*“As of now, it’s been almost a month without metformin, so, the sugar levels for many patients are becoming high. Those who can afford to buy are going to buy, whilst others are not buying, they are just staying.” **HCW2, Female, 62 years***

Unavailability of diabetes medication at the hospital resulted in missing doses for some clients or under-dosing:

*“Yes, sometimes I do stay without taking my diabetes medication. If I don’t find the medication here at the hospital, and I have no money, that’s it, there is nothing.” **person with diabetes 8, Female, 66 years***
*“For example, if I am prescribed metformin 500mg and the pharmacy doesn’t have 500mg tablets, but 850mg. In that case, I will be given the 850mg pills to break into two, which doesn’t give the correct measurement. Or if they prescribe 1g, but the pharmacy only has 850mg tablets, you are forced to take the 850mg, which again is less than the prescribed dosage.” **person with diabetes 18, male, 64years***
*“For those of us on insulin, I see that it is always available. I have never gone back without medication. The only problem I encounter are the syringes. Sometimes they are not available, and other times the type available is not durable, they easily bend.” **person with diabetes 17, Male, 57 years***

We observed that DAM assisted people with diabetes in purchasing drugs that were not available at the hospital pharmacy from private pharmacies that were charged less to help them cut costs. On each clinic day, a representative from DAM announced that there was no metformin at the hospital pharmacy, and asked those who were on metformin and needed help to be given money, and they would go buy for everyone.

***Theme 3: Healthcare worker’s knowledge, attitudes and skills.*** The lack of adequate knowledge of diabetes by some healthcare workers at health facilities has contributed to delays in the diagnosis of diabetes. Often, clients make multiple visits to health facilities with complaints specific or not specific to diabetes and would only be treated for the symptoms without getting a blood glucose test. The lack of diagnosis consequently affects the initiation of diabetes treatment and necessary lifestyle changes.

*“As for me I started feeling unwell around four years ago… I was feeling weak; my mouth was dry. When I went to hospital I was admitted and treated for anemia… after some time I was admitted at another hospital for vitamin B12 deficiency… but although I was receiving treatment, I was still feeling unwell… then one day a year ago, I got so weak I could not even climb stairs at work… when I got here (QECH), they said I needed a referral letter from a health center… I could not go back to the health center near where I stay, so I went to another clinic close to the referral hospital… When they checked my sugar, it was 523mg.” **person with diabetes 20, Male, 57 years***

In some instances, a diagnosis was made only after the onset of diabetes complications, despite multiple visits to a health facility.

*“In Malawi to have a diagnosis sometimes the system can fail you... For example, I met a client who had peripheral neuropathy, she had been to private hospitals and other clinics but her sugar was never tested… when I checked her sugar it was high and simply started her on glibenclamide [an antidiabetic medication].” **HCW4, Male, 45 years***

Fragmentation of clients’ care and a lack of a holistic approach to care also contributed to missing opportunities for diabetes diagnosis, hence delaying the commencement of treatment. One participant narrated how she used to have visual problems but was only being treated for them without having her blood glucose checked.

*“When I started taking medication and following what I was told, after about three months I noticed change. The things I was experiencing stopped. My eyes started to see better, because then I was using glasses since I was in secondary school. I had been changing the glasses often saying they were a problem. But after I started taking medication I came to a point where I did not need the glasses anymore.” **person with diabetes 01, Female, 67 years***

Limited knowledge of diabetes care for some healthcare workers and a lack of family involvement also contributed to the mismanagement of clients.

*“Sometimes I feel that healthcare workers have the mentality that a lay person cannot tell them anything… at one point I was taken to hospital because my sugar was high… my son wanted to tell the nurses that I am diabetic, but they asked him to stay away. . . then they put me on a glucose drip. When I regained consciousness, I pulled it off, and they thought I was mentally disturbed”. **person with diabetes 03, Female, 37 years***
*I found a diabetic patient being given glibenclamide and insulin together which is not supposed to be given like that… it’s not every healthcare worker who knows about diabetes. **HCW1, Female, 57 years***

***Theme 4: Diabetes health education and awareness.*** Many participants perceived the health education provided at the clinic to be helpful in empowering them to manage themselves effectively. For some, health education was the main reason for attending the QECH diabetes clinic rather than private or other public health facilities.

*“Healthcare workers always teach us on how to take care of our patients, and they keep giving us new information on caring for people with diabetes.” **FM8, female, 35 years***
*“As for me, I used to attend a private clinic. But all they were doing was to check your records, and prescribe your diabetes medication, but no counselling… then my daughter advised me ‘you need to go to a government hospital because there, there is education, once you learn, we will support you’. . .” **P2FGD3, Male, 65 years***
*“I had been getting my medication at a private clinic for two years... Its only when I started coming to QECH that I understood diabetes self-management. For all the years I was going to the private hospital, it was just about receiving medication then go home. It’s only now that I know the food restrictions... And since then, I feel healthy.” **P5FGD4, Male, 64 years***

There were delays in offering health education on diabetes after diagnosis. People newly diagnosed with diabetes were told to return on the next available diabetes clinic day to attend detailed lessons. Therefore, many people spent the first few days of their diagnosis without knowing how to manage themselves appropriately.

Below is what one family member said regarding the diagnosis of her husband.

*“I cannot explain anything [about diabetes] because this is my first time here. He only got diagnosed with diabetes two weeks ago… three days ago when we came for review, his sugar was high and we were admitted overnight and told to come today. Today we stayed long at the laboratory waiting for results…Unfortunately, when we got here, we found that the health education had finished” **FM7, Female, 40** years*

This is what the diabetes nurses had to say regarding when education is given:

*“They (newly diagnosed) are then booked to come for review after two weeks and that’s when they receive the full package of diabetes education.” **HCW1, Female, 57 years***

Although diabetes health education services were available at the health facility, participants noted that these services were also necessary for the general community. Many people with diabetes explained that their first time hearing about diabetes was when they were diagnosed with the disease. Below is what one participant narrated during the FGDs.

*“The other problem here in Malawi is that although there are many diabetic people, there is no civic education to sensitize people about the disease... At the moment, education is only for those who have diabetes, yet they were not taught the signs and symptoms of diabetes before they got sick.” **P6FGD3, Male, 44 years***

Similarly, many family members of people with diabetes expressed that their first time learning about diabetes was when their relationship was diagnosed with the disease.

*I did not have any idea about diabetes until my mother got sick. **FM8, Female, 35 years***
*I wish there were community awareness campaigns on diabetes. As for us, we wasted time going to a traditional healer before coming to the hospital because we didn’t know about diabetes. **FM5, Female, 34 years***

Opportunities for peer education also exist in QECH. The Diabetes Association of Malawi (DAM) is a formal social network that provides peer education and treatment support for people with diabetes. Through the initiative of the diabetes association, health education for people with diabetes was introduced at QECH as follows:

*“Previously, there were no diabetes lessons here at the clinic. So, we started asking each other what is diabetes? we started searching for information and we used the information were getting from other sources... and we were also using our experiences to understand what diabetes is... So, I think the doctors heard that we were conducting some diabetes lessons by ourselves, they took advantage of that and said ‘no! no! I think we should also start education’. . .” **person with diabetes 18, Male, 64 years***

However, there are divergent views between people with diabetes and healthcare workers regarding peer education. Participants that were DAM (Diabetes Association of Malawi, a patient group) members felt that the clinic is not utilizing the trained peer educators well enough:

*“One of the challenges we are facing now is that healthcare workers are not providing opportunities for peer education. Instead of letting us teach one another and ask one another questions, they take over. . .Now the World Diabetes Foundation is encouraging peer education and even the International Diabetes Federation is also encouraging peer education.” **person with diabetes 18, Male, 64 years***
*“We always have challenges especially with peer education. The aim of International Diabetes Federation is to promote peer education, between patients for them to easily understand each other. I live with diabetes, if I am to educate someone about diabetes, I will base it on my own experiences. Whereas if someone does not have diabetes, they just read about it, they will teach what’s in the books”. **person with diabetes 19, Male, 61** **years***

On the contrary, some healthcare workers had opposing views on peer education:

*“They are volunteers at the Diabetes Association and they work hand in hand with the clinic staff, to fill whatever gap there may be. I tell them to teach if I am not available and they came prepared but the teaching is different from ours. Ours is better so we prefer to teach by ourselves*.” **HCW1. Female, 57 years**
*“I think that most of the times when members of the association teach, there are certain things which they don’t know better, so the ones who teach better are the nurses. Of course, some members of the association are trained diabetes educators, but still more because they are not healthcare workers, it’s only the health workers who know most of the things and they are the ones who teach very well.” **HCW2, Female, 62 years***

Some participants described that on the diabetes clinic days, people also formed informal networks sharing experiences of living with diabetes and how they manage themselves. These meetings usually occurred when the clients were seated outside the clinic waiting for the FBG results. Unfortunately, it was observed that sometimes other people with diabetes discouraged their colleagues from following healthy dietary habits, as advised at the hospital.

*“And one other thing that influence us a lot are the discussions we have when we sit out there waiting for results... Some would say “as for me I eat anything. Those things if you take them serious it’s when you get more sick. Your body dries up, and you die faster”. And another would say “let’s be honest, imagine you are at a wedding and everyone is receiving Fanta, should you refuse?” … that’s where we disturb each other. We hear some advice which is confusing. When you get home you start to think “the way that one looks yet she eats anything, is there a problem?” you start to get carried away …” **P9FGD2, Female, 52 years.***
*“Someone once said to me “my friend, if you are too strict with following these instructions, you will get wasted. Because it’s not as if the disease will get away from you today. Sometimes try to eat a little bit of other things”. This was from someone who actually attends the education sessions with us here” **P6FGD3, Male, 44 years***

However, there are no existing guidelines or protocols to guide health education delivery. There were differences in how the lessons were delivered among the HCW in terms of content and duration.

*“Everyone has got their own different teaching techniques. The way my colleagues teach is not the same way I teach, we differ. I teach the whole package of diabetes” **HCW1, Female, 57 years***
*“It is hard for us to teach every topic on its own because we have new people attending every clinic day so it necessary that they learn everything, otherwise, their next clinic visit will only be after three months **.” HCW2, Female, 62 years***
*“Each one of us have their own way of teaching. We were told teaching should only take for 30 minutes. You should not teach foot care, diet, complications, and so forth at once because people lose concentration.” **HCW3, Female, 55 years***

***Theme 5: Glucose monitoring, prevention and management of complications.*** Most people with diabetes rely on the hospital to check their blood glucose on diabetes clinic days, which they attend once every three to five months. Healthcare workers, however, felt that FBG alone was not adequate for monitoring glucose control and suggested testing for glycated hemoglobin (HbA1C).

*“The way diabetes is managed in Malawi, I honestly don’t know how to describe it but it’s like you are operating in the dark. You say ‘what’s the fasting blood sugar of this particular patient?’ and maybe it’s 150 but does that tell you a true picture of how the sugars are being controlled? What do the international guidelines say about glycemic control? Ok, you have to go by glycated hemoglobin because that will give you an average blood glucose level for that particular patient. It’s normal to see patients cheating their way to the doctor to say ok, ‘I am going to discipline myself for the last three or four days before the clinic so that my blood glucose should be normal.... A very interesting thing is, you see that the patient who has been described to be having good blood good sugar developing diabetic complications, what should that be? So, we need to do something about it. I know that is something that will attract some resources, but we need to do that.”* **HCW6, Male, 35 years old**
*“I think the existing protocols are quite adequate for our setting, but I think what can be included are things that are not in our setting for example taking HbA1C which is a better way of monitoring sugar levels.” **HCW8, Female, 26 years old***

Many people with diabetes with high glucose levels attributed it to psychological stress.

*“What I realized is that whenever I receive sad news, my sugar goes up. Same way it happens with people with high blood pressure.” **PLWS03, Female, 37 years old***
*“Of course, I follow the instruction but maybe it’s because lately I have been thinking too much and travelling a lot which has made my sugar to be high.” **people with diabetes 5, Female, 61 years old***
However, there were no stress management protocols at the diabetes clinic, as observed by one of the healthcare workers.
*“I think there isn’t a proper referral system for stress management. I have never seen people referring patients but if someone has got poor adherence we actually say ‘nurses could you sit down and find out more from them’ but not specifically for stress … I don’t think there is any stress management in the clinic.” **HCW4, Male, 45 years***

Participating healthcare workers and people with diabetes also felt that the clinic was not adequately monitoring clients to prevent diabetes complications as it used to happen previously.

*“In the past, everyone was being reviewed by a doctor. And they would assess us thoroughly…. They would ask you to give urine sample for testing. This doesn’t happen anymore. We are just living ignorantly*.” **P9FGD1, Female, 39 years**
*“In the past, after we give health education, we would check everyone’s feet. If there are skin cracks in between toes, then we advise on those cracks. If we notice there is a sore, we help them. That’s what we are supposed to do. Previously, we would have time to say “let everyone take off their shoes”. If they are wearing inappropriate shoes, we’d advise them not to wear such shoes.”* **HCW3, Female, 55 years**
*“The second thing is checking the cholesterol levels is also very important because we know that diabetes is also associated with, increase circulation of low density lipoprotein especially the cholesterol. And in that case we have an increased risk of vascular complications, in particular, neuropathy, retinopathy and nephropathy. But also larger vessels like atherosclerosis, strokes and acute coronary syndromes. We are not doing that as at the moment. And as clinicians, we feel there is a huge gap.” **HCW6, Male, 35 years***

Several male participants also felt that the clinic did not address their complaints related to erectile dysfunction as a complication of diabetes. Lack of privacy at the clinic also prohibited clients from reporting issues related to sexual dysfunction, as consultations with nurses were conducted in an open area.

*“No, I have never reported this problem… the thing is, when we are reviewed by nurses, the line moves fast. But if you have a problem, they say you should go to see a doctor. And when I think of the time to go up there and wait for my turn to meet the doctor, I just think of going back home.” **person with diabetes 08, Male, 66 years***

In addition, male participants with diabetes defined wellness not only by their glucose levels but also by having their erectile dysfunction resolved. Below is what one participant had to share.

*“Although my blood sugar is mostly within normal ranges, I don’t feel fine, because I have not regained my strength as a man, I hope you know what I mean…Most of the time when I complain about that problem [erectile dysfunction], the response is always ‘aah, you come back another day.’. . .” **person with diabetes 17, Male, 57 years***

## Discussions

In this study, we investigated the perceptions of service users (people with diabetes and their family members) and HCW regarding the three dimensions of quality of care (structure, process, and outcome) at the QECH diabetes clinic in Blantyre Malawi. Previous studies at the QECH diabetes clinic have reported high rates of diabetes complications among clinic attendees, which is an outcome measure of the quality of care. We expounded on this knowledge by seeking perceptions of people with diabetes, their family members, and healthcare workers regarding the quality of care at the clinic to understand how it influences diabetes outcomes. We found that the quality of diabetes care at the QECH was perceived to be mixed, with some things going well and others not.

People with diabetes and their family members perceived some of the care at the QECH diabetes clinic to be of good quality. In particular, they appreciated the availability of free services including laboratory tests, diabetes education lessons, consultations and medications, linkages to peer support, and staff commitment at the clinic. Public healthcare services in Malawi are free at the point of care. However, due to erratic drug supply, including insulin syringes at hospital pharmacies, out-of-pocket expenditure at private pharmacies is often necessary. Consequently, some individuals with diabetes reported having missed their glucose control medications if they had no money. Medication stockouts could also discourage others from attending clinics, as drug refill is one of the main reasons people come to the clinic. Access to medications, diagnostic tests, and health education is important for the delivery of high-quality care to people with diabetes. Studies from some Sub-Saharan African countries, such as Kenya, Mali, and Nigeria, have shown that the economic burden of diabetes on individuals is substantial and affects the quality of care ^33^. Additionally, studies in high-income countries such as the United States of America have reported that people living in poverty and lacking healthcare insurance receive low-quality diabetes care ^34, 35^.

Quality of care was also perceived as low because of access challenges. This was possibly due to the organization of diabetes healthcare services (structure), whereby diabetes clinics were only available once a week at a tertiary level and not at primary care facilities, contributing to the suboptimal quality of care, which frustrated the people with diabetes. The structure created an access barrier, as some people with diabetes spent more time and transported money to the facility. The centralization of care also resulted in a high patient load for healthcare workers; therefore, clients had a long waiting time to get services, and healthcare workers could not thoroughly assess all clients. On each clinic day, there were two to four nurses and two to four doctors and/or clinical officers on duty, who attended to about 150 people. According to clinic records, the QECH diabetes clinic had 2470 registered clients in 2012 and operated twice a week ^36^. At the time of this study, the clinic conducted the study once a week with over 4,400 registered patients. Our findings regarding long distances to the diabetes clinic and long waiting times are similar to those found at another public referral hospital in central Malawi ^37^, suggesting that this could be a common problem in the country.

The quality of diabetes care was also compromised owing to incomprehensive and irregular assessments. The high patient load also made glucose monitoring irregular (once in 4 to 5 months), as the laboratory could only process up to 150 fasting blood glucose samples on each diabetes clinic day. The lack of regular glucose monitoring and other tests, such as complete cholesterol testing, worried doctors as it limited them from initiating treatment to prevent diabetes complications such as heart diseases. Another concern was the finding that foot assessment for all clients attending the clinic was no longer being performed regularly because time was not enough for the few available healthcare workers. A previous study on the challenges of foot care at QECH reported that the consequence of not doing foot assessments at the clinic was that foot ulcers are only identified when they are at an advanced stage and lead to sepsis and limb amputations ^38^. A cross-sectional study reporting diabetes treatment coverage using data from 55 low- and middle-income countries found that less than 10% of people with diabetes reported receiving comprehensive diabetes care according to the WHO guidelines and recommendations ^39^.

Both service users and HCW cited some instances of mismanagement of people with diabetes by HCW. The lack of adequate knowledge of diabetes by HCW in Malawi has also been reported in other studies ^40, 41^. This could be due to the lack of adequate training for some HCW in diabetes management and the lack of reference materials with detailed information to guide management at diagnosis or during follow-up care. The available treatment guidelines mostly focused on acute care, offered limited guidance for ongoing self-management support, and did not include management of complications, such as erectile dysfunction and emotional support ^42, 43^. Attending to the psychosocial needs of people with diabetes is important for achieving glycemic control and preventing diabetic complications ^44^. In particular, depression among people with diabetes is high (41%) in Malawi ^45^. The WHO recommends the development of national evidence-based protocols and standards for diabetes as vital tools for improving care ^46^. Countries with national guidelines on diabetes management that are updated regularly have achieved substantial improvements in client-centered care that is accepted by all stakeholders ^44, 47– 49^.

Opportunities for maximizing peer support through already established structures at the QECH Diabetes Clinic exist. However, there are mixed views between service providers and service users regarding the utility of peer education. While people with diabetes felt that peer education was important and that the educator provided information based on a lived experience, the healthcare workers were of the view that areas of emphasis differ depending on who is offering the education. The benefits of peer support structures extend beyond health education alone. DAM members at QECH were observed to assist in the purchase of medications that were not available at the hospital pharmacy, which helped people with diabetes save time, transport costs, and reduced prices by purchasing in bulk. Studies from other countries have shown evidence that peer support also promotes glycemic control and prevents depression ^50^. Therefore, collaboration between healthcare workers and DAM should be encouraged to maximize the benefits of existing peer support structures.

One limitation of our study was that it was hospital-based; hence, the participants could not articulate issues related to accessibility of services in the same way as those who did not come to the clinic at all. Second, data were collected at only one public central hospital; therefore, perspectives of the HCW may not be similar to those at facilities of different natures. However, since the participating clients were from both urban and rural settings, it is likely that their perceptions regarding the quality of care at other public health facilities in Malawi could be similar. Therefore, the study findings provide important insights into areas that need attention to enhance the healthcare system’s capacity for the quality of diabetes care.

## Conclusion

The quality of care in diabetes clinics is perceived to be mixed. Although service users appreciated the available services, they noted that the clinics were difficult to access, overcrowded, and understaffed; hence, care was not comprehensive. A shortage of glucose control medications was common and compounded on these issues. HCW observed that the available treatment guidelines were not comprehensive in diabetes management, which resulted in inconsistent care practices. We recommend allocating more staff at diabetes clinics and strengthening primary care facilities by offering in-service training in diabetes management, providing point-of-care diagnostic equipment, and stocking the right mix of medication so that services are easily accessible and comprehensive. Good quality diabetes care services are essential for improving the well-being of people with diabetes.

## Ethical consideration and consent

The research process complied with the ethical principles of the World Medical Association’s (WMA) ethical principles on research involving human subjects as stipulated in the Declaration of Helsinki ^51^ and as required by the Constitution of the Republic of Malawi Bill of Rights, chapter IV ^52^. Ethics approval for the study was obtained from the College of Medicine Research and Ethics Committee (approval number: P.08/17/229) of Kamuzu University of Health Sciences on 4 ^th^ October 4, 2017. Participation in the study was voluntary, and all participants signed an informed consent form available in both English and Chichewa before enrolment in the study. For participants who could not read the questionnaire, the researcher read the study details. Participants who could not write or sign used a thumbprint to provide consent. All participants were made aware of their right to withdraw at any point, without reprisal.

## Data Availability

Interview guides and anonymized transcript data can be accessed as extended data https://doi.org/10.6084/m9.figshare.28845914.v1 ^53^. Figshare: Extended data – quality of diabetes care at QECH. https://doi.org/10.6084/m9.figshare.28845914.v1 ^53^ This project contains the following extended data: Individual in depth interview guide for people with diabetes Focus group discussion guide for people with diabetes Individual in depth interview guide for family members Individual in depth interview guide for healthcare workers Consolidated criteria for reporting qualitative research (COREQ) Anonymized transcripts from individual IDIs, FGDs and KIIs The data are available under the terms of the Creative Commons Attribution 4.0 International license (CC-BY 4.0).
