# Supplementary material for: 'Operating in the dark’: perspectives of health service users and healthcare workers on quality of diabetes care at a public referral hospital in Blantyre, Malawi - a qualitative study
Source: Wellcome Open Res. 2025 May 16;10:241. [Version 1] doi: 10.12688/wellcomeopenres.21374.1 (PMC12645092; doi:10.12688/wellcomeopenres.21374.1)
Supplement: Peer review history

# Reviewer report: Reviewer response for version 1

**DOI:** 10.21956/wellcomeopenres.23639.r139279

**Published:** 2025-11-24

**Authors:** Jazlan Jamaluddin

**Affiliations:** 1 Universiti Malaya, Kuala Lumpur, Federal Territory of Kuala Lumpur, Malaysia

This study provides important insights into perceptions of diabetes care in a low resource setting. The use of qualitative interviews and focus groups is appropriate, and the authors present meaningful themes supported by participant quotations. The manuscript is generally well structured and clearly written. However, several areas within the methods, analysis and reporting require clarification or could be strengthened by acknowledging limitations more explicitly.

- The paper briefly mentions the lead researcher’s interest in diabetes care but gives little detail on how her personal views or role in the health system may have influenced the study. Suggest to acknowledge this in the limitations and add a short reflexive note in the methods.
- It is unclear whether the researcher knew participants beforehand or how participants perceived her.
- Suggest to note this gap in the limitations and briefly state the nature of researcher–participant relationships in the Methods.
- The study does not report who declined to participate or whether participants differed from non participants. Family members were recruited by convenience, and only clinic attenders were included, excluding those who dropped out of care. Suggest to address this as a sampling limitation and add a short explanation in the methods.
- Focus groups were held during busy clinic days, likely favouring participants with more free time or better health.
- Suggest to recognise this as a contextual limitation in the limitations section.
- Although male and female groups were separated, there is no discussion of how gender, age or hierarchy may have influenced participation. Suggest to add a short reflection in the methods or limitations about possible group dynamics.
- Using a structured guide can reduce flexibility and restrict deeper exploration. Suggest to note this as a methodological limitation and explain why the structured guide was chosen.
- There is no explanation of whether questions changed as new ideas emerged. Suggest to clarify in the methods whether guides stayed fixed or changed, or acknowledge this as a limitation.
- The study does not fully explain how initial codes were generated or whether a codebook was refined. Suggest to add brief clarification in methods or identify this as a limitation.
- The analysis does not state whether opposing or minority views were considered. Suggest to acknowledge this in the limitations as a potential analytic gap.
- Manual coding is acceptable, but there is little explanation of how decisions were documented beyond mentioning an audit trail. Suggest to add a short statement in the methods or address this in the limitations.
- Issues like erectile dysfunction and stress were raised, but the clinic setting may have made participants less comfortable discussing them in depth. Suggest to acknowledge this as a limitation linked to the interview environment.

Is the work clearly and accurately presented and does it cite the current literature?

Yes

If applicable, is the statistical analysis and its interpretation appropriate?

Yes

Are all the source data underlying the results available to ensure full reproducibility?

Yes

Is the study design appropriate and is the work technically sound?

Yes

Are the conclusions drawn adequately supported by the results?

Yes

Are sufficient details of methods and analysis provided to allow replication by others?

Partly

Reviewer Expertise:

Endocrine Disorder, Primary Care, Family Medicine

I confirm that I have read this submission and believe that I have an appropriate level of expertise to confirm that it is of an acceptable scientific standard.

# Reviewer report: Reviewer response for version 1

**DOI:** 10.21956/wellcomeopenres.23639.r124646

**Published:** 2025-08-23

**Authors:** Anand Krishnan

**Affiliations:** 1 All India Institute of Medical Sciences, New Delhi, India

The study looks at the critical issue of diabetes care in developing countries using a qualitative approach.

 The authors have done well to describe the process of patient consultation in the study setting as that provides the right context to interpret the findings.

 It might be good to include in the results from different perspectives- what was good or positive aspects and what were the negative aspects. At present it seems to be heavily skewed to the negative side with almost no positive aspects. Is that a correct reflection of the “truth”? some comments are made in discussion section on the positive aspects.

 Ultimately the key issues identified – logistics & supplies, waiting time and inconvenience, lack of provider knowledge and patient education are generic health system issues applicable to most diseases including acute conditions. The last one is more specific for chronic diseases and diabetes. It would be good to comment on this in the discussion.

Is the work clearly and accurately presented and does it cite the current literature?

Yes

If applicable, is the statistical analysis and its interpretation appropriate?

Not applicable

Are all the source data underlying the results available to ensure full reproducibility?

No source data required

Is the study design appropriate and is the work technically sound?

Yes

Are the conclusions drawn adequately supported by the results?

Yes

Are sufficient details of methods and analysis provided to allow replication by others?

Yes

Reviewer Expertise:

NA

I confirm that I have read this submission and believe that I have an appropriate level of expertise to confirm that it is of an acceptable scientific standard.
